# Laryngeal adenoid cystic carcinoma: case report

**DOI:** 10.1590/S1516-31802007000500010

**Published:** 2007-09-02

**Authors:** Edson Ichihara, Alfio José Tincani, Albina Altemani, Antônio Santos Martins

**Keywords:** Adenoid cystic carcinoma, Larynx, Salivary gland, Radiotherapy, Laryngectomy, Carcinoma adenóide cístico, Laringe, Glândulas salivares, Radioterapia, Laringectomia

## Abstract

**CONTEXT:**

Adenoid cystic carcinomas are malignant tumors that occur in both the major and the minor salivary glands. A laryngeal location is rare because of the paucity of accessory salivary glands in this area. Adenoid cystic carcinomas account for less than 1% of all malignant tumors in the larynx, and only about 120 cases have been reported in the literature. These tumors have a slight female predisposition, and their peak incidence is in the fifth and sixth decades of life. In this article, we describe a case of laryngeal adenoid cystic carcinoma and discuss its clinical characteristics and treatment.

**CASE REPORT:**

We report on a case of laryngeal adenoid cystic carcinoma in a 55 year-old female patient who presented with dyspnea and hoarseness. Features of the diagnostic and therapeutic evaluation are described and the clinical management of such cases is outlined. The clinical course, definitive treatment strategy and surgical procedure, and also adjuvant treatment with irradiation are discussed. Although the tumor is radiosensitive, it is not radiocurable.

## RESUMO

**CONTEXTO:**

Carcinoma adenóide cístico (CAC) é um tumor maligno que ocorre tanto nas glândulas salivares maiores quanto nas menores. Localização laríngea é rara devido à paucidade de glândulas salivares acessórias nesta região. O carcinoma adenóide cístico representa menos de 1% das lesões malignas da laringe e apenas 120 casos foram relatados na literatura. O CAC tem freqüência discretamente superior no sexo feminino e seu pico de incidência ocorre entre a quinta e sexta décadas de vida. Neste artigo, descrevemos um caso de CAC laríngeo e discutimos suas características clínicas e seu tratamento.

**RELATO DE CASO:**

Relatamos o caso de CAC numa paciente de 55 anos que apresentava disfonia e dispnéia. Características diagnósticas e avaliação terapêutica são descritas e a conduta clínica definida. Evolução clínica, estratégia terapêutica e procedimento cirúrgico são discutidos, bem como o tratamento adjuvante com radioterapia. O tumor, apesar de radiossensível, não é radiocurável.

## INTRODUCTION

Adenoid cystic carcinomas are malignant tumors that occur in both the major and the minor salivary glands. A laryngeal location is rare because of the paucity of accessory salivary glands in this area. Adenoid cystic carcinomas account for less than 1% of all malignant tumors in the larynx, and only about 120 cases have been reported in the literature until now.^1,2^ Two-thirds of these laryngeal tumors occur in the subglottis.^3^

These tumors have a slight female predisposition, and their peak incidence is in the fifth and sixth decades of life.^4^ There is no distinct risk factor that predisposes patients towards this malignancy.^4^ Patients usually present with a history of mild to severe dyspnea at diagnosis, and histological confirmation is obtained during tracheotomy to treat tumors that are up obstructed airways.

## CASE REPORT

A 55 year-old woman who was a former smoker was referred to our service with an 18-month history of progressive dyspnea and hoarseness. The patient had no history of cough or dysphagia. Fiber bronchoscopy detected a vegetative lesion in the posterior commissure of the larynx that extended downwards to the trachea. During clinical investigation, the patient developed acute respiratory failure, which was treated as community-acquired pneumonia. On physical examination, she showed inspiratory laryngeal stridor.

Fiberoptic laryngoscopy showed an endophytic lesion in the subglottic area with laryngeal stenosis of 80%. Tracheotomy was performed under local anesthesia and a lesion biopsy obtained. The pathology report revealed that the specimen was consistent with an adenoid cystic carcinoma of the larynx.

Further investigation using esophagoscopy did not show any suspicious lesions, although there was difficulty in introducing the esophagoscope through the left piriform sinus. Computed tomography (CT) confirmed the presence of a submucosal mass in the subglottic area. Neither physical examination nor neck CT detected any evidence of node involvement. The findings from chest X-ray were normal.

We performed total laryngectomy without neck dissection and left thyroid lobectomy with isthmusectomy. Additionally, the lower margin of the third tracheal ring was sent for frozen-section biopsy, which did not show any evidence of disease.

**Figure 1:**
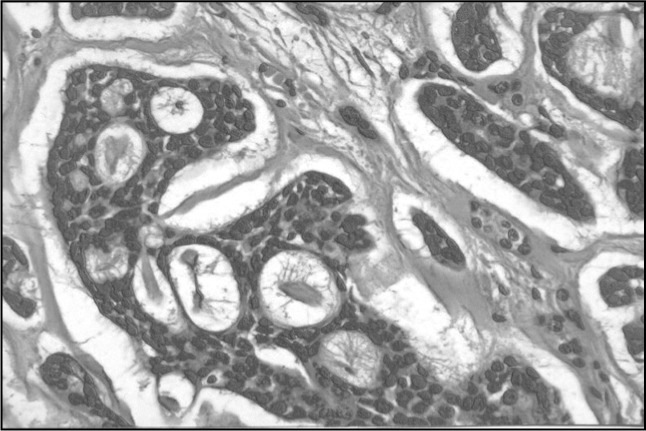
Histological section of laryngeal adenoid cystic carcinoma. (400 x magnification and hematoxylineosin staining).

The final pathology report showed negative margins in the subglottis, cricoid cartilage and tracheal ring. The tumor affected both the vocal folds and the posterior commissure and invaded both the left thyroid lobe and the extralaryngeal soft tissues of the neck, with a maximum width of 2.5 centimeters. Although there was no lymphatic spreading, perineural invasion (which is a feature of adenoid cystic carcinoma) was grossly present (Figure 1).

## DISCUSSION

Adenoid cystic carcinoma usually occurs as a largely asymptomatic, nonulcerated submucosal mass. As a result, diagnosis is often delayed and, in the larynx, subglottic tumors have the opportunity to invade deeply before they are diagnosed.

Preoperative histopathological analysis is essential because the symptoms do not differ greatly from squamous cell carcinoma. Neck metastasis is rare, occurring in only 10 to 15% of the cases.^1^ Since early perineural and hematological spreading is common, local recurrences and distant metastases (especially to the lung) are common and sometimes arise years after the primary tumor has been diagnosed and treated. Therefore, these patients require long-term follow-up.

An extensive review of the literature revealed that there have been about 120 well-documented cases of adenoid cystic carcinoma of the larynx. Morais Pérez et al.^2^ reported only 80 cases up to 1999, and after that year we found only another 40 cases reported in the literature. According to Dexemble et al.,^3^ 64% of laryngeal adenoid cystic carcinoma cases occur in the subglottis, 25% in the supraglottis, 5% in the glottis and 6% in the transglottic area. The first case of hypopharyngeal adenoid cystic carcinoma was reported in 2004 by Kufeld et al.^5^

The histopathological pattern of adenoid cystic carcinoma is classified into three distinct subtypes: cribriform, which is the most common; tubular, which has the best prognosis; and solid, which carries the worst prognosis.^4^

As is true of adenoid cystic carcinoma originating elsewhere, distant metastases are occasionally stable and slow-growing. Therefore, with aggressive surgery, the five-year survival rates for patients with laryngeal adenoid cystic carcinoma have been reported to range from only 12 to 17%.^1–3,6^

In view of the rarity of laryngeal adenoid cystic carcinoma, the treatment options are still controversial. Previous case reports contain different management strategies and few data on follow-ups. Yet most authors agree that the treatment of choice is wide-margin local excision (partial or total laryngectomy, depending on the location and size of the tumor).^1–3,5,6^ In the absence of neck metastasis, elective neck dissection is unnecessary. Radical neck dissection is indicated for patients who have clinically or histologically confirmed nodal metastases.^1–6^

The role of radiotherapy is still open to debate. These tumors have been shown to be radiosensitive but usually not radiocurable. Therefore, radiotherapy alone usually has little role in treatment.^1,6^ It is possible that radiotherapy may cure adenoid cystic carcinoma in a prepubescent larynx,^1^ but this carries the risk of causing a condition of fibrotic infantile larynx and leaving the patient with no useful voice, airway or swallowing function.

Since our patient had a large tumor with perineural invasion, she was scheduled for adjuvant radiotherapy.

## CONCLUSION

Laryngeal adenoid cystic carcinoma is a rare entity and, in many cases, the diagnosis is only made when the disease has become advanced. Therefore, a high degree of suspicion is essential for early diagnosis.

In our opinion, although other treatment options have been suggested, the best tumor management includes wide-margin surgery with postoperative radiotherapy for advanced lesions that present perineural spreading or close or positive margins.

For these patients, long-term follow-up is essential because of the possibility of late hematological metastasis.
